# Regional localisation of p53-independent apoptosis determines toxicity to 5-fluorouracil and pyrrolidinedithiocarbamate in the murine gut

**DOI:** 10.1038/sj.bjc.6603224

**Published:** 2006-06-27

**Authors:** S P Bach, S E Williamson, S T O'Dwyer, C S Potten, A J M Watson

**Affiliations:** 1 Nuffield Department of Surgery, University of Oxford, Level 2, John Radcliffe Hospital, Oxford OX3 9DU, UK; 2 Paterson Institute, Wilmslow Road, Manchester M20 4BX, UK; 3 Department of Surgery, Christie Hospital National Health Service Trust, Wilmslow Road, Manchester M20 4BX, UK; 4 Epistem, The Incubator Building, 48 Grafton Street, Manchester M13 9XX, UK; 5 Gastroenterology Research Group, The Henry Wellcome Laboratory, Nuffield Building, University of Liverpool, Crown St, Liverpool L69 3BX, UK

**Keywords:** colorectal cancer, 5-fluorouracil, pyrrolidinedithiocarbamate, *N*-acetylcysteine, p53, apoptosis

## Abstract

Pyrrolidinedithiocarbamate (PDTC) enhanced the activity of 5-fluorouracil (5-FU) in a colorectal cancer xenograft model. Pyrrolidinedithiocarbamate also reduced gastrointestinal toxicity associated with 5-FU therapy in large but not small bowel. We sought to clarify the basis of this differential enteric toxicity. Apoptosis and mitosis were assessed on a cell positional basis in small and large intestinal crypts of p53 wild-type (+/+) and p53 null (−/−) mice 6, 12, 24, 36, 48 and 72 h after the administration of high (200 mg kg^−1^) or low (40 mg kg^−1^) dose 5-FU±250 mg kg^−1^ PDTC. Regimens were chosen to model a single human dose and a weekly schedule. The effects of another antioxidant *N*-acetylcysteine (NAC) were also investigated. Large intestinal crypts affect apoptosis purely by p53-dependent mechanisms, whereas small intestinal crypts are able to initiate both p53-dependent and -independent pathways following treatment with 5-FU. Pyrrolidinedithiocarbamate and NAC antagonised p53-dependent but potentiated p53-independent apoptotic activity. Consequently, the proportion of surviving clonogens increased in the large but not in the small intestine. Regional availability of p53-dependent and -independent apoptotic pathways in small and large intestine together with separate modulation of these pathways by antioxidants explains the different regional enterotoxicity following 5-FU therapy.

The dose-limiting enteric side effects of 5-fluorouracil (FU) are due to a profuse wave of p53-driven apoptosis within normal intestinal crypts accompanied by prolonged cell cycle arrest (Pritchard *et al*, 1998). The antioxidants pyrrolidinedithiocarbamate (PDTC) and *N*-acetylcysteine (NAC) have been shown to enhance the activity of 5-FU against colorectal cancer cell lines independent of p53 function (Chinery *et al*, 1997; Bach *et al*, 2001). This is important as p53 is frequently mutated in human cancer (Levine *et al*, 1991). Although PDTC increased 5-FU-induced apoptosis within tumour xenografts through the induction of p21^WAF1/CIP1^ via CCAAT/enhanced binding protein*β* (Chinery *et al*, 1997), gastrointestinal toxicity was unexpectedly reduced (Bach *et al*, 2000). Pyrrolidinedithiocarbamate significantly improved colonic clonogen survival following the administration of 5-FU, whereas small intestinal toxicity remained unchanged. This may be of biological significance as 5-FU was a factor of 1 log more toxic to large, compared to small intestinal crypts. Similar relative toxicities have previously been reported in humans (Milles *et al*, 1962). Reduced apoptosis among colonic clonogens and prompt restoration of normal mitotic activity appeared to account for the reduction in drug-induced toxicity. Separate investigators have independently demonstrated the ability of antioxidant compounds to ameliorate the toxicity of 5-FU and a range of other chemotherapeutic compounds, including oxaliplatin and paclitaxel (Alexandre *et al*, 2006b).

Reactive oxygen species (ROS) are unstable molecules derived from normal mitochondrial metabolism. They set about obtaining additional electrons from other molecules, such as DNA, proteins and cell membranes. As a result of these interactions, donors may incur structural damage that has led to the classical conception of ROS as toxic metabolites. Antioxidants such as PDTC are electron donors that have cytoprotective properties in disease states that induce oxidative stress such as liver cirrhosis (Bruck *et al*, 2002) and transplant reperfusion injury (Ross *et al*, 2000; Tsuchihashi *et al*, 2003).

Good evidence now suggests that ROS also act as physiological signalling molecules in a number of cellular processes including proliferation and apoptosis. In this context, antioxidants such as NAC exert an antiproliferative effect (Sekharam *et al*, 1998; Nargi *et al*, 1999; Menon *et al*, 2003; Kang *et al*, 2004). This is of special interest given that ROS production may be essential for the growth of p53-deficient colorectal tumours (Sablina *et al*, 2005).

A third emerging view of antioxidants is as cellular markers for apoptosis or necrosis following cytotoxic drug treatment. Generation of H_2_O_2_ was consistently detected following treatment with camptothecin, vinblastine, inostamycin and adriamycin in a lung tumour cell line (Simizu *et al*, 1998). Scavenging of H_2_O_2_ caused failure of apoptosis. Reactive oxygen species generation has been demonstrated in a range of tumours 6–48 h after treatment with various anticancer agents (Ikeda *et al*, 1999; Huang *et al*, 2003; Fawcett *et al*, 2005; Rahmani *et al*, 2005). Indeed, the antitumour activity of paclitaxel in mice was abolished by NAC as accumulation of H_2_O_2_ is an early and crucial step for paclitaxel-induced cancer cell death (Alexandre *et al*, 2006a).

In this study, we sought to clarify the basis of differential small and large bowel toxicity following combined treatment with PDTC and 5-FU. We determined the availability of p53-dependent and -independent apoptotic pathways in the small and large intestine following 5-FU therapy and characterised the effect of PDTC upon each pathway. We hypothesised that 5-FU would initiate activity in both types of pathway but that separate modulation by PDTC at this level might account for the differences in small and large intestinal clonogenic stem cell survival. We measured p53-dependent and -independent apoptosis in the small and large intestine of p53 wild-type and knockout mice 6, 12, 24, 36, 48 and 72 h after high- or low-dose 5-FU therapy administered with or without PDTC. We also determined the effects of a separate antioxidant, NAC, upon 5-FU-induced apoptosis in the small and large intestine.

## MATERIALS AND METHODS

### Animals

p53 wild-type (+/+) and p53 null (−/−) mice were from a colony housed at the Paterson Institute for Cancer Research, Manchester, UK derived from those generated by Donehower *et al* (1992). Mice were aged 10–12 weeks, weighed approximately 25 g and were kept under a 12 h light, 12 h dark regimen, with free access to water and the RM1-expanded diet (Special Diet Services, Waltham, Essex, UK). Experiments were performed in accordance with the Animals (Scientific Procedures) Act 1986 and revised UKCCCR guidelines (1997).

### Materials

5-Fluorouracil (Roche, Welwyn Garden City, UK) was administered at 5 mg ml^−1^ or 25 mg ml^−1^ depending upon dose. Pyrrolidinedithiocarbamate (Sigma, Poole, UK) and NAC (Sigma, Poole, UK) were freshly prepared in 0.9% (wt/vol) saline to a concentration of 6.25 mg/0.2 ml. Drug administration was by bolus intraperitoneal injection with the first injection given at 0900 hours. Pyrrolidinedithiocarbamate or NAC were administered 15 min before 5-FU. Optimum times for apoptotic assessment were determined from previous studies (Pritchard *et al*, 1997; Pritchard *et al*, 1998; Bach *et al*, 2000).

### Effects of treatment upon apoptosis and mitosis in the small and large intestine

Apoptosis and mitosis were assessed on a cell positional basis in small and large intestinal crypts of p53 wild-type (+/+) and p53 null (−/−) mice 6, 12, 24, 36, 48 and 72 h after administration of (a) low-dose 5-FU (40 mg kg^−1^)±250 mg kg^−1^ PDTC and (b) high-dose 5-FU (200 mg kg^−1^)±250 mg kg^−1^ PDTC. The effects of another antioxidant NAC were determined in wild-type and null mice 24 and 48 h after administration of high-dose 5-FU (200 mg kg^−1^)±200 mg kg^−1^ NAC.

The rationale for choosing 40 mg kg^−1^ 5-FU is that it models a single human dose (Pritchard *et al*, 1997). The LD_50_ for 5-FU in the mouse is reported to be in the order of 450 mg kg^−1^ (Burns and Beland, 1984). We have corroborated this figure for the p53 knockout mouse (Bach, 2003). The higher dose of 200 mg kg^−1^ 5-FU is equivalent to 600 mg m^−2^ in this mouse, the exact concentration of 5-FU employed for weekly combination schedules with folinic acid in humans (Buroker *et al*, 1994). PDTC (250 mg kg^−1^ ) was the maximum tolerated as a single dose in previous experiments utilising the BDF1 mouse (Bach *et al*, 2000), whereas 200 mg kg^−1^ NAC approximates to the clinical dose used in humans (Prescott *et al*, 1989).

Mice were culled by cervical dislocation and intestinal specimens promptly harvested. Six mice were utilised per experimental group at each time point. Control animals, injected with saline (0.2 ml), were culled at the first time point. Small and large intestine were prepared by methods described in detail previously (Merritt *et al*, 1996). Apoptotic bodies and mitotic figures were easily identified in haematoxylin- and eosin-stained sections by their characteristic morphological appearance. We validated morphological scoring of apoptosis against the terminal deoxynucleotide transferase labelling (TUNEL) technique (ApopTag; Oncor, Gaithersburg, MD, USA) (Bach, *et al* 2000). Statistically valid results can be obtained by examining 200–300 half-crypt sections from 4–6 mice, per group (Chwalinski and Potten, 1989). Good cross-sections cut through the centre of a crypt's longitudinal axis were examined using light microscopy (magnification × 640). Each crypt was considered as two-halves, divided along the luminal axis with cells numbered sequentially from the crypt base. Each position was scored as containing a normal cell, an apoptotic cell (with one or more apoptotic bodies) or a mitotic figure. Data were recorded using a PC microcomputer and software developed at the Paterson Institute (Ijiri and Potten, 1983). Group treatment was masked and a single observer (SPB) scored the slide sections. For each cell position, apoptotic and mitotic indices were calculated as percentages. Data may be pooled over a series of adjacent cell positions enabling comparisons to be made between stem, presumed clonogenic and transitional cell zones within the crypt. Results were analysed by a computer program developed at the Paterson Institute based on the median test, a nonparametric ranking test (Potten *et al*, 1990). *P*-values less than 0.001 were considered significant.

### Detection of p21^WAF1/CIP1^

Intestinal samples were stained for p21^WAF1/CIP1^; ab5 (Oncogene, San Diego, CA, USA). Positive controls: irradiated mouse enterocytes demonstrate nuclear staining. Negative controls: substitution of nonimmune serum for primary antibody on a second tissue section. Antigen retrieval was achieved by microwaving at high power (800 W) for 20 min. Primary antibody concentrations of 1 : 200 were determined by serial dilutions. Samples were processed using the avidin–biotin complex immunoperoxidase staining system (Santa Cruz Biotechnology, Santa Cruz, CA, USA), developed with diaminobenzidine and counterstained with thionine blue.

## RESULTS

A five-fold increase in the dose of 5-FU administered from 40 to 200 mg kg^−1^ did not dramatically affect either the kinetics of onset of apoptosis, the magnitude of the apoptotic response or the cell positional incidence of apoptosis within small or large intestinal crypts of wild-type animals. Dose escalation did considerably extend the period of mitotic suppression. We shall describe results obtained with 200 mg kg^−1^ in detail (data obtained following administration of 40 mg kg^−1^ 5-FU can be found in supplementary files). Administration of PDTC as a single agent had no effect upon apoptotic or mitotic indices (data not shown).

### Colon

#### Wild type

**Figure 1:**
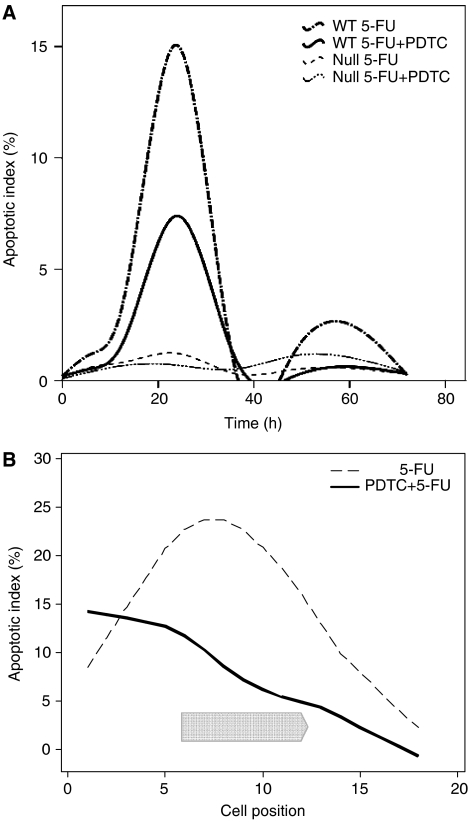
Large intestinal apoptosis. (**A**) Time course of mean apoptotic index is displayed for the whole crypt (cell positions 1–18) following 200 mg kg^−1^ 5-FU alone±250 mg kg^−1^ PDTC in p53 wild-type and p53 null mice. Six animals per group at each time point (6, 12, 24, 36, 48 and 72 h) counting 50 half-crypts per mouse. (**B**) Apoptotic index is displayed for each crypt cell position 24 h after the administration of treatment in p53 wild-type mice. Chevron indicates cell positions over which a significant difference was detected between treatments (*P*<0.001, PC crypts).

5-Fluorouracil increased apoptosis in p53 wild-type large intestinal crypts with maximal values at 24 h in the presumed clonogenic stem cell compartment (cell positions 4–9) (Figure 1A and B). Normal values were restored by 36 h. Following coadministration of PDTC, significantly less apoptosis occurred at 24 h (Figure 1A and B). Clonogenic and transit cells were reprieved (cell positions 6–13, *P*<0.001). *N*-acetylcysteine also inhibited apoptosis at 24 h (Table 1).

**Figure 2:**
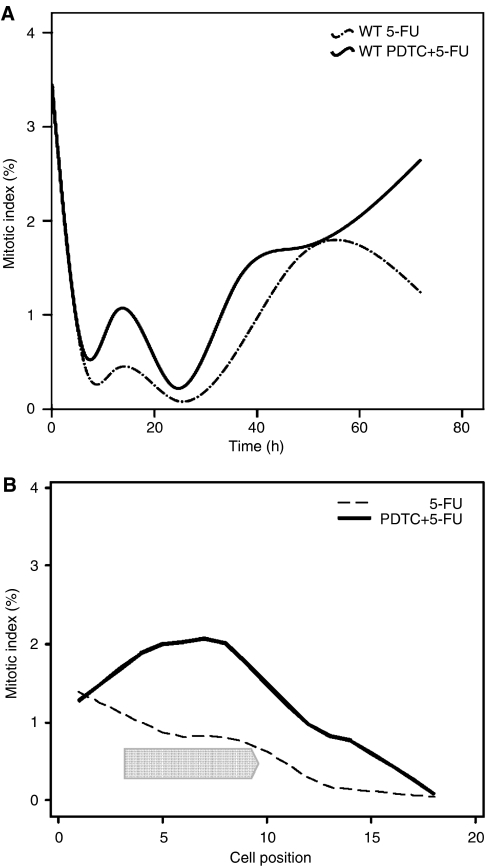
Large intestinal mitosis. (**A**) Time course of mean mitotic index is displayed for the whole crypt (cell positions 1–18) following 200 mg kg^−1^ 5-FU alone±250 mg kg^−1^ PDTC in p53 wild-type and p53 null mice. Six animals per group at each time point (6, 12, 24, 36, 48 and 72 h) counting 50 half-crypts per mouse. (**B**) Mitotic index is displayed for each crypt cell position 72 h after the administration of treatment in p53 wild-type mice. Chevron indicates cell positions over which a significant difference was detected between treatments (*P*<0.001, PC crypts).

Administration of 5-FU produced a prompt decrease in the mitotic index at 6 h with a complete cessation of almost all measured activity by 24 h (Figures 2A). Recovery remained incomplete at 72 h. Coadministration of PDTC facilitated early restoration of normal mitotic activity among clonogenic stem cells at 72 h (cell positions 4–9, *P*<0.001) (Figure 2B).

#### Null

No significant elevation in apoptotic values occurred within p53 null colonic crypts throughout the entire 72-h period following 5-FU±PDTC (Figure 1A) or NAC (Table 1). 5-Fluorouracil decreased the mitotic index at 6 h with only partial recovery by 72 h (data not shown). The addition of PDTC did not significantly alter these parameters.

### Small intestine

#### Wild type

**Figure 3:**
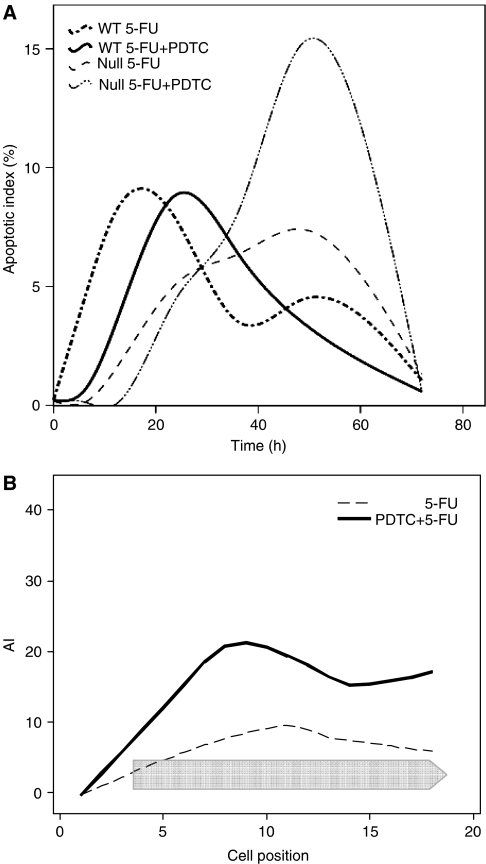
Small intestinal apoptosis. (**A**) Time course of mean apoptotic index is displayed for the whole crypt (cell positions 1–18) following 200 mg kg^−1^ 5-FU alone±250 mg kg^−1^ PDTC in p53 wild-type and p53 null mice. Six animals per group at each time point (6, 12, 24, 36, 48 and 72 h) counting 50 half-crypts per mouse. (**B**) Apoptotic index is displayed for each crypt cell position 48 h after the administration of treatment in p53 null mice. Chevron indicates cell positions over which a significant difference was detected between treatments (*P*<0.001, PC crypts).

5-Fluorouracil alone increased apoptosis in p53 wild-type small intestinal crypts with maximal values occurring between 12 and 24 h within the clonogenic stem cell compartment (Figure 3A and Supplementary data). The addition of PDTC delayed the onset of apoptosis from 6 to 12 h across all compartments with peak values seen later between 24 and 36 h (Figure 3A). *N*-acetylcysteine increased apoptosis at 48 h (Table 1).

5-Fluorouracil produced a prompt decrease in the mitotic index at 6 h with recovery at 72 h (data not shown). Coadministration of PDTC did not modulate these parameters (data not shown).

#### Null

5-Fluorouracil alone produced a modest elevation in apoptosis above baseline values at the 12, 24, 36 and 48 h time points (Figure 3A). The addition of PDTC delayed the onset of apoptosis beyond 12 h (Figure 3A), and by 48 h, peak values were double than those observed with 5-FU alone throughout the whole crypt axis (cell positions 3–18, *P*<0.001) (Figure 3B). Similarly, elevated peak values were seen following NAC (Table 1). Peak values equated to those measured in similarly treated wild-type animals. This late rise in apoptosis within p53 null small intestinal crypts was also seen following 40 mg kg^−1^ 5-FU+PDTC at 36 h; cell positions 3–13 (*P*<0.001) (Supplementary data).

5-Fluorouracil produced a prompt decrease in the mitotic index at 6 h with complete cessation of almost all measured activity up to 72 h at which point recovery occurred (data not shown). Coadministration of PDTC did not modulate these parameters (data not shown).

### p21^WAF1/CIP1^ expression in small intestinal crypts

**Figure 4:**
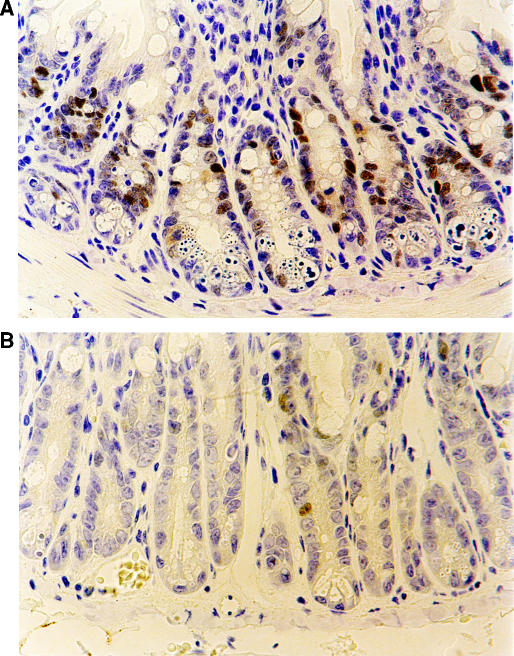
Immunohistochemical sections from (**A**) p53+/+ (× 400) and (**B**) p53−/− mouse small intestinal crypts (× 500) stained for p21^WAF1^ 48 h following 200 mg kg^−1^ 5-FU and 250 mg kg^−1^ PDTC.

No p21^WAF1/CIP1^ expression occurred in controls (saline injection) (data not shown). Elevated nuclear expression of the p21^WAF1/CIP1^ protein occurred within the transit cell population 6 h after administration of PDTC±5-FU (data not shown) with maximal staining intensity in surviving cells at 48 h (Figure 4A), resolving by 72 h. Nuclear staining was determined on a cell positional basis at 48 h with high labelling indices among animals treated with 5-FU±PDTC concentrated about cell positions 6–10 and no statistically significant difference between groups (data not shown).

Despite achieving high levels of apoptosis within small intestinal crypts of p53 null mice treated with PDTC/5-FU, no significant induction of p21^WAF1/CIP1^ occurred at any time point (Figure 4B).

## DISCUSSION

### Large intestinal toxicity

In p53 wild-type large intestine, administration of 5-FU alone produced an isolated apoptotic peak in all crypt compartments at 24 h (Figure 1A). Coadministration of PDTC and NAC significantly reduced the number of apoptotic events measured at 24 h, especially among clonogenic stem cells (Figure 1B). Apoptotic activity in p53 null colon remained at baseline values throughout the 72 h period regardless of whether PDTC or NAC were also administered (Figure 1A). Pyrrolidinedithiocarbamate facilitated early mitotic recovery in the wild-type colon at 72 h (Figure 2B), whereas recovery at this time point remained incomplete in mice treated with 5-FU alone (Figure 2B). In summary, the murine large intestine initiated only p53-driven apoptosis in response to 5-FU. These events were inhibited by both PDTC and NAC producing a marked improvement in the proportion of surviving clonogens. Mitotic recovery was subsequently enhanced and tissue regeneration as measured by the crypt survival assay improved (Bach *et al*, 2000).

### Small intestinal toxicity

In p53 wild-type small intestine, 5-FU initiated a rise in apoptosis within 6 h; peak values occurred between 12 and 24 h (Figure 3A). Pyrrolidinedithiocarbamate did not simply antagonise maximal apoptotic values as occurred in the large intestine. At 6 and 12 h, PDTC reduced apoptosis among stem, presumed clonogenic and transit cell populations (Figure 3A and Supplementary data). The situation was subsequently reversed as PDTC then led to higher apoptotic values compared to 5-FU alone at 24 and 36 h (Figure 3A). Higher apoptotic values were observed at 48 h following administration of 5-FU and NAC (Table 1). No difference in mitotic recovery was seen in the wild-type small intestine following PDTC. A combination of reduced clonogen availability and prolonged p21^WAF1/CIP1^ expression will limit mitotic recovery under these circumstances (Pritchard *et al*, 1998).

In contrast to results from p53 null large bowel, increased apoptotic activity was recorded in the p53 null small intestine following 5-FU administration. It was also notable that dose escalation from 40 to 200 mg kg^−1^ 5-FU produced a markedly increased the magnitude and duration of apoptotic activity (Supplementary data). At 200 mg kg^−1^ 5-FU, peak apoptotic values in the p53 null small intestine approached those seen in wild types, although these values occurred somewhat later in null littermates, between 24 and 48 h (Figure 3A). Pyrrolidinedithiocarbamate and NAC significantly augmented peak levels of p53-independent apoptosis at 36 and 48 h by a factor of two to three (Figure 3A and B and Table 1). Despite finding abundant p21^WAF1/CIP1^ expression within wild-type small intestinal crypts at these time points (Figure 4A), no expression was detected within p53 null enterocytes (Figure 4B). This is in contrast to a previous report utilising PDTC and 5-FU therapy in p53 null colorectal cancer cell lines where PDTC was found to induce apoptosis via p21^WAF1/CIP1^ (Chinery *et al*, 1997). In our model, augmentation of p53-independent apoptosis by PDTC does not result from modulation of p21^WAF1/CIP1^. Administration of PDTC did not alter the duration of cell cycle arrest as mitotic recovery occurred simultaneously for both groups at 72 h (data not shown).

It appears that small intestinal crypts can affect programmed cell death by both p53-dependent and -independent pathways following treatment with the antimetabolite 5-FU. We are unable to specify what proportion of late apoptotic events are mediated by p53-dependent and -independent pathways respectfully in the wild-type mouse, although it is clear from their null littermates that appreciable p53-independent activity is possible and that this activity is upregulated by coadministration of PDTC or NAC. As a result, PDTC does not significantly modulate the number of surviving clonogens in the small intestine; mitotic recovery is not expedited and consequently this agent has no impact upon small intestinal crypt survival (Bach *et al*, 2000).

### Molecular control of intestinal toxicity

The mechanism by which 5-FU induces apoptosis in the normal intestine involves initial interference of RNA signalling pathways followed by activation of the p53 pathway (Pritchard *et al*, 1997; Bunz *et al*, 1999; Longley *et al*, 2002). Several transcriptional targets of p53 are genes whose products generate ROS (Polyak *et al*, 1997; Macip *et al*, 2003). It is hypothesised that these products contribute towards p53-mediated apoptosis. In addition, a set of 5-FU-dependent, p53-inducible genes, continue to be defined. In the context of our study, the most interesting of these is a gene coding for mitochondrial ferredoxin reductase (FDXR). Ferredoxin reductase may represent a mechanism by which ROS are generated. Reactive oxygen species cause permeabilisation of the mitochondrial membrane with release of the proapoptotic proteins cytochrome *c* and apaf-1 (Kroemer and Reed, 2000). Ferredoxin reductase expression following 5-FU exposure in a p53+/+ CRC cell line correlated with increased oxidative stress and apoptosis (Hwang *et al*, 2001). Increasing levels of FDXR independent of 5-FU therapy did not result in increased apoptosis (Liu and Chen, 2002). Ferredoxin reductase contains a p53-responsive element in its promoter region (Liu and Chen, 2002). The effects of 5-FU were abrogated in FDXR knockout clones and pharmacological inhibition of 5-FU activity was achieved by the use of two antioxidant agents, ubiquinol and a mitochondrial-targeted derivative mitoQ (Hwang *et al*, 2001). MitoQ was shown to prevent the reduced clonogenicity associated with 5-FU treatment *in vitro* in accordance with our results using PDTC and 5-FU *in vivo* (Bach *et al*, 2000). A mechanism therefore exists to explain the observed reduction in p53-driven apoptosis following the addition of PDTC or NAC to 5-FU therapy.

Apoptosis does not feature in the response of p53 null colonic enterocytes to 5-FU (Figure 1A). Similar effects have been reported in this model following radiation treatment (Merritt *et al*, 1994) and in a colorectal cancer cell line subject to 5-FU (Bunz *et al*, 1999). Interestingly, the five most highly transcriptionally activated genes in a CRC cell line following 5-FU exposure all possess putative p53 response elements (Maxwell *et al*, 2003). It seems likely that the unspecified p53-independent apoptotic mechanism present within small intestinal enterocytes and augmented by PDTC is simply absent from colonocytes.

In conclusion, it appears that large intestinal crypts affect apoptosis purely by p53-dependent mechanisms, whereas small intestinal crypts are able to initiate both p53-dependent and -independent pathways following treatment with 5-FU. Pyrrolidinedithiocarbamate and NAC antagonised p53-dependent but potentiated p53-independent apoptotic activity. Consequently, the proportion of surviving clonogens increased in the large but not in the small intestine and early recovery of normal mitotic activity occurred only in the large bowel. Functional outcome at a tissue level is measured in terms of improved large bowel crypt regeneration 6 days after an enterotoxic schedule of 5-FU (Bach *et al*, 2000). Despite the fact that PDTC modulates murine large bowel toxicity to 5-FU in isolation, this finding may still be of clinical relevance. We have previously shown that 5-FU is relatively more toxic (by a factor of one log) to large as opposed to small intestinal crypts in our mouse model (Bach *et al*, 2000). Evidence exists to suggest that this situation may also be replicated in humans (Milles *et al*, 1962). Pyrrolidinedithiocarbamate or NAC in addition to other agents with similar biochemical properties may therefore reduce the p53-driven apoptotic events that mediate 5-FU-related drug-induced toxicity. p53-independent apoptotic pathways that are likely to be recruited therapeutically were not similarly affected. The potential to reduce diarrhoea during multimodality neoadjuvant therapy for rectal cancer should also be examined further.

## Figures and Tables

**Table 1:** Large and small intestinal apoptotic activity in p53 wild-type and p53 null mice following high dose 5-FU (200 mg kg^−1^)±NAC (200 mg kg^−1^)

		**Apoptotic index (%)**			
		**Large intestine**		**Small intestine**	
**p53**	**Time (h)**	**5-FU**	**5-FU+NAC**	**5-FU**	**5-FU+NAC**
Wild-type	Control	0.21	0.21	0.22	0.22
	24	15.05	**6.40** a	**7.80**	**8.21**
	48	1.10	0.33	**4.39**	**6.20** a
					
Null	Control	0.09	0.09	0.18	0.18
	24	1.11	0.72	**5.12**	**6.68**
	48	0.48	0.42	**5.39**	**10.93** a

Apoptotic indices are expressed for the crypt as a whole over cell positions 1–18. Six animals were used in each group, counting 50 half-crypts per mouse. Control values (saline injection) are shown in ‘normal typeface’ as are treatment indices that do not significantly differ from these baseline values (*P*>0.001, PC crypts). Treatment indices outside control values are displayed in bold (*P*<0.001, PC crypts).

a A significant difference between 5-FU and 5-FU/NAC treatments at a specific time point (*P*<0.001, PC crypts).
