# Supplementary material for: Regional localisation of p53-independent apoptosis determines toxicity to 5-fluorouracil and pyrrolidinedithiocarbamate in the murine gut
Source: Br J Cancer. 2006 Jun 27;95(1):35–41. doi: 10.1038/sj.bjc.6603224 (PMC2360501; doi:10.1038/sj.bjc.6603224)
Supplement: Supplemental data [file 95-6603224x1.doc]

***SUPPLEMENTAL* Table 1 Small Intestinal Apoptotic Activity – Low Dose 5-FU**

| **p53** | **Time (hours)** | **Stem**  **(3-5)** | | **Clonogenic**  **(7-9)** | | **Transit**  **(11-13)** | | **Total**  **(1-18)** | |
| --- | --- | --- | --- | --- | --- | --- | --- | --- | --- |
|  |  | **5-FU** | **PDTC** | **5-FU** | **PDTC** | **5-FU** | **PDTC** | **5-FU** | **PDTC** |
| **Wildtype** | Control | 0.79 | 0.79 | 0.33 | 0.33 | 0.00 | 0.00 | 0.22 | 0.22 |
|  | 6 | **§9.33** | **2.30** | **6.07** | **3.11** | 0.48 | 0.92 | 3.93 | 1.37 |
|  | 12 | **10.44** | **10.71** | **6.88** | **12.09** | 0.85 | **1.56** | 4.53 | **5.71** |
|  | 24 | **7.38** | **§21.70** | **13.21** | **§20.15** | **6.06** | **3.33** | **6.60** | **11.50** |
|  | 36 | 1.08 | 1.34 | 0.54 | 2.19 | 0.14 | 0.00 | 0.61 | 0.81 |
|  | 48 | 0.81 | 0.40 | 0.40 | 0.40 | 0.41 | 0.14 | 0.39 | 0.32 |
|  | 72 | 2.75 | 0.34 | 0.67 | 1.01 | 0.42 | 0.22 | 1.23 | 0.47 |
|  |  |  |  |  |  |  |  |  |  |
| **Null** | Control | 0.54 | 0.54 | 0.00 | 0.00 | 0.00 | 0.00 | 0.18 | 0.18 |
|  | 6 | 0.00 | 0.27 | 0.13 | 0.13 | 0.00 | 0.00 | 0.11 | 0.14 |
|  | 12 | **§2.03** | 0.13 | **§1.62** | 0.00 | **§2.90** | 0.00 | **1.94** | 0.02 |
|  | 24 | **1.48** | **2.62** | **2.30** | **3.72** | **1.90** | 0.83 | **1.93** | 1.83 |
|  | 36 | **1.60** | **§6.23** | **1.33** | **§4.32** | 0.67 | **§3.59** | 0.91 | **3.92** |
|  | 48 | 0.81 | 0.67 | 0.13 | 0.40 | 0.13 | 0.27 | 0.33 | 0.39 |
|  | 72 | 0.17 | 0.83 | 0.68 | 0.66 | 0.17 | 0.16 | 0.48 | 0.53 |

Small intestinal apoptotic activity in p53 wildtype and null mice following low dose 5-FU (40mg/kg) plus/minus PDTC (250mg/kg). Apoptotic indices are expressed for the crypt as a whole over cell positions 1 to 18. Separate values are given for the stem cell (3-5), presumed clonogenic (7-9) and transit cell (10-12) compartments. Six animals were used in each group, counting 50 half crypts per mouse. Control values (saline injection) are shown in ‘normal typeface’ as are treatment indices that do not significantly differ from these baseline values (p>0.001, PC Crypts). Treatment indices outside control values are displayed in **bold** (p<0.001, PC Crypts). **§** indicates a significant difference between 5-FU and 5-FU/PDTC treatments at a specific timepoint (p<0.001, PC Crypts).

***SUPPLEMENTAL* Table 2** **Large Intestinal Apoptotic Activity – Low Dose 5-FU**

| **p53** | **Time (hours)** | **Stem**  **(1-3)** | | **Clonogenic**  **(7-9)** | | **Transit**  **(11-13)** | | **Total**  **(1-18)** | |
| --- | --- | --- | --- | --- | --- | --- | --- | --- | --- |
|  |  | **5-FU** | **PDTC** | **5-FU** | **PDTC** | **5-FU** | **PDTC** | **5-FU** | **PDTC** |
| **Wildtype** | Control | 0.11 | 0.11 | 0.34 | 0.34 | 0.11 | 0.11 | 0.21 | 0.21 |
|  | 6 | 0.68 | 1.21 | **2.57** | **1.76** | 0.71 | 1.07 | 1.14 | 1.30 |
|  | 12 | 0.68 | 1.77 | **2.57** | **2.05** | 0.71 | 0.96 | 1.17 | 1.15 |
|  | 24 | **§8.80** | **2.41** | **§20.81** | **13.01** | **§12.03** | **6.06** | **12.17** | **6.62** |
|  | 36 | 1.12 | 0.40 | 1.01 | 0.27 | 0.57 | 0.27 | 0.66 | 0.34 |
|  | 48 | 1.01 | 0.27 | **2.16** | 0.40 | 0.90 | 0.00 | 0.96 | 0.20 |
|  | 72 | 0.22 | 0.40 | 0.22 | 1.77 | 0.11 | 0.27 | 0.15 | 0.79 |
|  |  |  |  |  |  |  |  |  |  |
| **Null** | Control | 0.00 | 0.00 | 0.17 | 0.17 | 0.00 | 0.00 | 0.09 | 0.09 |
|  | 6 | 0.17 | 0.68 | 0.50 | 0.40 | 0.50 | 0.54 | 0.42 | 0.39 |
|  | 12 | - | 0.13 | - | 0.13 | - | 0.13 | 0.60 | 0.11 |
|  | 24 | 0.00 | 0.67 | 0.34 | 0.27 | 1.01 | 1.21 | 0.42 | 0.60 |
|  | 36 | - | 0.40 | - | 0.40 | - | 0.40 | 0.40 | 0.38 |
|  | 48 | - | 0.13 | - | 0.53 | - | 0.13 | 0.10 | 0.35 |
|  | 72 | - | 0.13 | - | 0.27 | - | 0.13 | 0.16 | 0.11 |

Large intestinal apoptotic activity in p53 wildtype and null mice following low dose 5-FU (40mg/kg) plus/minus PDTC (250mg/kg). Apoptotic indices are expressed for the crypt as a whole over cell positions 1 to 18. Separate values are given for the stem cell (1-3), presumed clonogenic (7-9) and transit cell (11-13) compartments. Six animals were used in each group, counting 50 half crypts per mouse. Control values (saline injection) are shown in ‘normal typeface’ as are treatment indices that do not significantly differ from these baseline values (p>0.001, PC Crypts). Treatment indices outside control values are displayed in **bold** (p<0.001, PC Crypts). **§** indicates a significant difference between 5-FU and 5-FU/PDTC treatments at a specific timepoint (p<0.001, PC Crypts).

***SUPPLEMENTAL* Table 3 Small Intestinal Apoptotic Activity – High Dose 5-FU**

| **p53** | **Time (hours)** | **Stem**  **(3-5)** | | **Clonogenic**  **(7-9)** | | **Transit**  **(11-13)** | | **Total**  **(1-18)** | |
| --- | --- | --- | --- | --- | --- | --- | --- | --- | --- |
|  |  | **5-FU** | **PDTC** | **5-FU** | **PDTC** | **5-FU** | **PDTC** | **5-FU** | **PDTC** |
| **Wildtype** | Control | 0.79 | 0.79 | 0.33 | 0.33 | 0.00 | 0.00 | 0.22 | 0.22 |
|  | 6 | **§5.27** | 0.56 | **§10.02** | 0.89 | **§4.00** | 0.90 | **4.46** | 0.62 |
|  | 12 | **§9.71** | **4.60** | **§16.50** | **6.24** | **§7.75** | **3.29** | **8.09** | **3.35** |
|  | 24 | **6.79** | **7.52** | **10.36** | **§17.19** | **7.69** | **§10.75** | **7.80** | **8.86** |
|  | 36 | **4.56** | **6.64** | **6.54** | **§9.82** | **2.03** | **§5.81** | **3.52** | **6.40** |
|  | 48 | **5.78** | **3.75** | **5.55** | **5.89** | **3.94** | **3.60** | **4.39** | **3.60** |
|  | 72 | 2.63 | 0.67 | 0.78 | 0.87 | 0.67 | 0.33 | 1.04 | 0.58 |
|  |  |  |  |  |  |  |  |  |  |
| **Null** | Control | 0.54 | 0.54 | 0.00 | 0.00 | 0.00 | 0.00 | 0.18 | 0.18 |
|  | 6 | 0.00 | 0.26 | 0.13 | 0.13 | 0.00 | 0.00 | 0.11 | 0.14 |
|  | 12 | 0.95 | 0.13 | 1.21 | 0.00 | **§1.93** | 0.00 | 1.48 | 0.02 |
|  | 24 | **5.14** | **6.40** | **9.88** | **7.58** | **§6.14** | **2.90** | **5.12** | **4.51** |
|  | 36 | **6.58** | **5.39** | **11.01** | **14.06** | **5.92** | **8.61** | **6.35** | **8.09** |
|  | 48 | **3.77** | **§8.41** | **7.30** | **§21.62** | **8.15** | **§18.45** | **5.39** | **15.06** |
|  | 72 | 1.01 | 0.66 | **2.02** | 0.66 | **2.03** | 0.16 | 1.31 | 0.55 |

Small intestinal apoptotic activity in p53 wildtype and null mice following high dose 5-FU (200mg/kg) plus/minus PDTC (250mg/kg). Apoptotic indices are expressed for the crypt as a whole over cell positions 1 to 18. Separate values are given for the stem cell (3-5), presumed clonogenic (7-9) and transit cell (10-12) compartments. Six animals were used in each group, counting 50 half crypts per mouse. Control values (saline injection) are shown in ‘normal typeface’ as are treatment indices that do not significantly differ from these baseline values (p>0.001, PC Crypts). Treatment indices outside control values are displayed in **bold** (p<0.001, PC Crypts). **§** indicates a significant difference between 5-FU and 5-FU/PDTC treatments at a specific timepoint (p<0.001, PC Crypts).

***SUPPLEMENTAL* Table 4 Large Intestinal Apoptotic Activity – High Dose 5-FU**

| **p53** | **Time (hours)** | **Stem**  **(1-3)** | | **Clonogenic**  **(7-9)** | | **Transit**  **(11-13)** | | **Total**  **(1-18)** | |
| --- | --- | --- | --- | --- | --- | --- | --- | --- | --- |
|  |  | **5-FU** | **PDTC** | **5-FU** | **PDTC** | **5-FU** | **PDTC** | **5-FU** | **PDTC** |
| **Wildtype** | Control | 0.11 | 0.11 | 0.34 | 0.34 | 0.11 | 0.11 | 0.21 | 0.21 |
|  | 6 | 1.47 | 0.33 | 2.16 | 0.45 | 0.67 | 1.13 | 1.19 | 0.52 |
|  | 12 | **5.04** | **3.57** | **4.38** | 1.70 | **2.51** | 0.78 | **2.91** | 1.42 |
|  | 24 | **10.96** | **13.64** | **§24.72** | **11.77** | **§17.20** | **5.68** | **15.05** | **7.39** |
|  | 36 | 0.90 | 1.59 | 0.90 | 1.93 | 0.68 | 0.67 | 0.62 | 1.06 |
|  | 48 | 0.90 | 0.45 | **§2.63** | 0.11 | 1.24 | 0.23 | 1.10 | 0.16 |
|  | 72 | 0.56 | 0.34 | 0.34 | 0.45 | 0.11 | 0.22 | 0.24 | 0.29 |
|  |  |  |  |  |  |  |  |  |  |
| **Null** | Control | 0.00 | 0.00 | 0.17 | 0.17 | 0.00 | 0.00 | 0.09 | 0.09 |
|  | 6 | - | - | - | - | - | - | 0.60 | 0.40 |
|  | 12 | - | - | - | - | - | - | 0.79 | 0.65 |
|  | 24 | 0.27 | 0.80 | **3.01** | 1.06 | 1.08 | 0.40 | 1.21 | 0.68 |
|  | 36 | 0.26 | 0.27 | 0.26 | 0.13 | 0.42 | 0.81 | 0.32 | 0.50 |
|  | 48 | 0.52 | 0.39 | 0.78 | **§3.18** | 0.00 | 0.93 | 0.48 | 1.09 |
|  | 72 | - | - | - | - | - | - | 0.29 | 0.36 |

Large intestinal apoptotic activity in p53 wildtype and null mice following high dose 5-FU (200mg/kg) plus/minus PDTC (250mg/kg). Apoptotic indices are expressed for the crypt as a whole over cell positions 1 to 18. Separate values are given for the stem cell (1-3), presumed clonogenic (7-9) and transit cell (11-13) compartments. Six animals were used in each group, counting 50 half crypts per mouse. Control values (saline injection) are shown in ‘normal typeface’ as are treatment indices that do not significantly differ from these baseline values (p>0.001, PC Crypts). Treatment indices outside control values are displayed in **bold** (p<0.001, PC Crypts). **§** indicates a significant difference between 5-FU and 5-FU/PDTC treatments at a specific timepoint (p<0.001, PC Crypts).
